# Primary Hypothyroidism in Patients Exposed to Therapeutic External Beam Radiation: Non-randomized Comparative Study

**DOI:** 10.7759/cureus.32170

**Published:** 2022-12-03

**Authors:** Jyoti Dane, Ramesh Arya, Sushil Gour, Prabhakar Gupta, Preety Jain, Anamika Kore, Abhijit Das, Rajesh K Maurya

**Affiliations:** 1 Radiation Oncology, Government Bundelkhand Medical College, Sagar, IND; 2 Radiation Oncology, Mahatma Gandhi Memorial Government Medical College, Indore, IND; 3 Radiation Oncology, Bharat Aluminium Company (BALCO) Medical Centre, Raipur, IND; 4 Community Medicine, Government Bundelkhand Medical College, Sagar, IND

**Keywords:** external beam radiation therapy, cancer patients, thyroid dysfunction, sub clinical hypothyriodism, radiation, hypothyroidism

## Abstract

Background

Cancer patients are not routinely assessed for thyroid function after external beam radiotherapy (EBRT) to the neck, despite hypothyroidism being a known side effect of EBRT. So, this study aimed to assess the incidence of hypothyroidism after therapeutic external beam radiotherapy to the neck and to determine the time for the development of hypothyroidism.

Methodology

A non-randomized prospective comparative study was done at a tertiary care center from April 2018 to September 2020. Any cancer patients who were euthyroid before radiotherapy and are planned to receive EBRT to the neck were included as cases, whereas controls were selected from the patients who were euthyroid before radiotherapy and were planned to receive EBRT to the site other than the neck. A total of 100 participants in each case and control group were selected. Data were collected on participants’ age, gender, primary tumor site, treatment modality, total radiation dose along with concurrent chemoradiation regimens. Details of blood chemistry including thyroid hormone levels were collected during the pre-radiation phase and post-radiation phase. After the completion of radiotherapy, both the patients and controls were followed up periodically at three months, six months, nine months, 12 months, and finally at 15 months post-radiation. Data were analyzed and interpreted to pursue defined objectives by using tables and graphs using Microsoft Excel and IBM SPSS, version 26.0 (Armonk, NY: IBM Corp.). The chi-square test was applied to find out the association of different variables with the development of hypothyroidism. P-values<0.05 were considered significant throughout.

Results

According to our findings, the incidence of hypothyroidism following external beam radiotherapy to the neck where radiation portals included a portion or the entire thyroid gland was 16% and 4%, when the radiation given to sites other than neck region. The difference in incidence between the case and control groups was found to be statically significant (p<0.05). However, it was found that age, gender, the primary tumor site, total radiation dose, and treatment modality had no significant effects on hypothyroidism development. The median time duration to become hypothyroid after EBRT was 12 months.

Conclusions

The monitoring of thyroid function should become a part of routine follow-up procedures in all cancer patients who receive neck radiation as part of their treatment.

## Introduction

Cancer is a large family of diseases that involve abnormal cell growth which has the potential to spread to other parts of the body [1]. The signs and symptoms can be either generalized or local symptoms. Cancer incidence and mortality are rapidly growing worldwide. There are various type of treatment modalities available for cancer treatment which includes surgery, chemotherapy, radiation treatment, hormonal treatment, targeted therapy, and palliative treatment. However, treatment modality depends on the type, grade, severity, and location of cancer as well as the patient's health and performance status.

The effect of radiotherapy (RT) on thyroid function was first described in 1929 [2]. The most obvious development of hypothyroidism after external beam radiotherapy is observed among patients treated for Hodgkin’s disease [3]. Hypothyroidism is a known side effect of external beam radiotherapy (EBRT) to the neck including some portion or the entire thyroid gland. In addition to clinical hypothyroidism characterized by low free T4 and high thyroid stimulating hormone (TSH), this radiation effect can also cause subclinical hypothyroidism with normal free T4 and high TSH. Numerous studies have estimated the prevalence of subclinical hypothyroidism ranges from 3% to 15% [4]. In most cases, subclinical hypothyroidism develops into clinical hypothyroidism. Subclinical thyroid dysfunction is found more frequently and can be diagnosed by thyroid function tests, but these are often missed because routine testing of thyroid function tests is generally not done during follow-up.

It is recognized that hypothyroidism significantly impacts patients' quality of life. As the survival of cancer patients is increasing, the quantification of the incidence of radiation-induced hypothyroidism is necessary [5]. However, there is an underestimation and underreporting of radiation-induced hypothyroidism because the routine assessment of thyroid function is not done during follow-up, this will result in failure to identify and treat the reversible cause of morbidity for surviving patients [6].

The majority of publications on thyroid problems following radiation treatment are primarily retrospective. The availability of prospective studies is very less. Hence, our study aims to assess the incidence of hypothyroidism after therapeutic external beam radiotherapy to the neck, to determine the time for the development of hypothyroidism and the relation between the total radiation doses for the development of hypothyroidism.

## Materials and methods

Study design and setting

A non-randomized prospective comparative study was done at a tertiary care center, Government Cancer Hospital, Mahatma Gandhi Memorial Medical College, Indore, between April 2018 and September 2020.

Study participants

Cancer patients who are euthyroid before radiotherapy and are planned to receive EBRT to the neck where the radiation portals included a part or whole of the thyroid gland were included as cases, whereas for comparison purposes control were selected who are euthyroid before radiotherapy and are planned to receive EBRT to site other than neck.

The patients were included based on the following criteria: (1) patients of age above 18 years, (2) euthyroid patients, (3) patient's performance status according to Karnofsky Performance Score (KPS) >50% [7], (4) adequate renal function (creatinine <1.5 mg/dL), (5) adequate hepatic function (bilirubin <2 mg/dL, albumin >3.5 g/dL), and (6) adequate blood profile (Hb>10 g/dL, WBC >4000/mm^3^, platelets >1 lakh/mm^3^).

The patients were excluded based on the following criteria: (1) refusal to participate, (2) pregnant and lactating females, (3) diagnosed with thyroid dysfunction, (4) patients taking medications that impact thyroid function (tyrosine kinase inhibitors or immunotherapy), and (5) patients with associated comorbidity like uncontrolled hypertension, ischemic heart disease, diabetes mellitus, and pulmonary tuberculosis, etc.

Sample size and sampling

A total of 100 participants in each case and control group were selected. Non-probability sampling was done till the required sample size was achieved.

Study procedure and data collection

The conventional EBRT technique at various fractionation scheduled in cobalt 60 units was used in both the case and control groups. Data were collected on participants’ age, sex, primary tumor site, treatment modality, total radiation dose along with concurrent chemoradiation regimens. Details of blood chemistry, complete blood count, thyroid hormone levels (TSH, T3, T4 levels), abdominal ultrasonography, and chest x-ray were collected at the pre-radiation phase and post-radiation phase. After the completion of radiotherapy, both the patients and controls were followed up periodically at three months, six months, nine months, 12 months, and finally at 15 months post-radiation.

Thyroid function evaluation

Thyroid function was assessed by determining the levels of free T3 (normal values: 0.5 to 1.5 ng/dL), T4 (normal values: 5 to 15.0 g/dL), and TSH (normal values: 0.35 to 5.1 IU/mL) level. Though hypothyroidism can be classified as clinical/overt or subclinical, for the purposes of this study, we considered both subclinical and overt hypothyroidism as hypothyroidism.

Ethical considerations

This prospective study was approved by the Ethics and Scientific Review Committee, MGM Medical College and M Y Hospital, Indore (approval no.: EC/MGM/March-19/06). Informed written consent was taken from participants before enrolment and during the follow-up period, participants diagnosed with hypothyroidism were informed about their status and prescribed medication accordingly.

Data analysis

Data were analyzed and interpreted to pursue defined objectives by using tables and graphs using Microsoft Excel and IBM SPSS, version 26.0 (Armonk, NY: IBM Corp.) for Windows. Frequency and percentages were used to describe categorical variables. Inferential analysis was conducted using chi-square test for categorical variables. P-values of <0.05 was considered significant for the difference between various parameters among different groups.

## Results

Characteristics of study participants

Characteristics of Cases

The mean age of the participants in the cases group was 50 (±8.2) years. Among all the participants, 62 were males and 38 were females. Of all the cases, 76 were having head and neck cancer and rest 24 were having breast cancer. Carcinoma of buccal mucosa was the commonest among head and neck cancer with 19 cases, while carcinoma of the tongue was the second commonest (16 cases). Other cases of head and neck cancer included secondary neck, carcinoma of base of the tongue, larynx, alveolus, oropharynx, gingivobuccal sulcus, pyriform sinus, pyriform fossa, supraglottic and epiglottis.

Characteristics of Control

The mean age of the participants in the control group was 51.8 (±8.2) years. Among all the participants, 25 were males and 75 were females. A majority (72) of the participants in the control group was having cervical cancer, followed by prostate cancer (10), rectal cancer (7), carcinoma of the urinary bladder (6), vulval cancer (3), and carcinoma of the endometrium (2).

Incidence of hypothyroidism

Table 1 represents the incidence of hypothyroidism in the case and control groups. According to our study, the incidence of hypothyroidism after EBRT to the neck, where radiation portals cover all or part of the thyroid gland, was 16%. Among the control group the incidence of hypothyroidism after external beam radiotherapy was 4%. The difference in incidence between both groups was found to be statically significant (p<0.05).

**Table 1:** Incidence of hypothyroidism after EBRT. *Chi-square test. **Statistically significant. EBRT: external beam radiotherapy

Groups	Euthyroidism	Hypothyroidism	Total	p-Value*
Case	84	16	100	0.011**
Control	96	4	100	
Total	180	20	200	

Among the cases, a total of 16 patients developed hypothyroidism where 11 developed subclinical hypothyroidism and five patients developed clinical hypothyroidism. While in the control group four patients developed subclinical hypothyroidism but no one developed clinical hypothyroidism. However, age, gender, the location of the primary tumor, the total radiation dose, or the type of therapy did not significantly affect the onset of hypothyroidism (Table 2).

**Table 2:** Relationship between different variables and development of hypothyroidism among cases. ^*^Chi-square test. CCRT: concurrent chemoradiotherapy; RT: radiotherapy

Risk category	Subcategory	Overall	Euthyroidism	Hypothyroidism	p-Value*
Age (years)	<50	53	44 (83.02%)	9 (16.98%)	0.776
	≥50	47	40 (85.11%)	7 (14.89%)	
Gender	Male	62	55 (88.71%)	7 (11.29%)	0.117
	Female	38	29 (76.32%)	9 (23.68%)	
Tumor site	Breast	24	22 (91.67%)	2 (8.33%)	0.238
	Head and neck	76	62 (81.58%)	14 (18.42%)	
Radiation dose (Gy)	<45	24	22 (91.67%)	2 (8.33%)	0.238
	≥45	76	62 (81.58%)	14 (18.42%)	
Mode of treatment	RT	26	24 (92.31%)	2 (7.69%)	0.184
	CCRT	74	60 (81.08%)	14 (18.92%)	

Among the patients who developed hypothyroidism, only one patient developed hypothyroidism within three months, two patients in six months, three patients developed it within nine months, and five patients each developed hypothyroidism in 12 months and 15 months. The median time duration to become hypothyroid after EBRT was 12 months.

## Discussion

Our study assessed the incidence of hypothyroidism after therapeutic external beam radiotherapy to the neck. Among the patients who received EBRT to the neck, 16% developed hypothyroidism. While, among the patients who received EBRT to the site other than the neck, only 4% developed hypothyroidism. The incidence of hypothyroidism after neck irradiation was typically reported to range between 20% and 30% [8]. Our study found a 16% incidence, which was consistent with these results. For patients who have received radiotherapy to the neck region their association with hypothyroidism was found to be statically significant.

The incidence of hypothyroidism in male and female patients in our study was 11.29% (7/62) and 23.68% (9/38), respectively. The incidence of hypothyroidism was higher among patients who were below 50 years of age. Research has shown that women are more prone to hypothyroidism after neck irradiation than men [9]. However, neither age nor gender was found to be statistically significant in our study and it was persistent with the results of Mercado et al. and Bernát and Hrušák [10,11].

The role of radiation dose in hypothyroidism development remains controversial. Radiotherapy doses exceeding 60 Gy are associated with an increased risk of hypothyroidism among patients with head and neck cancer. However, previous studies showed that the radiation dose was not a significant factor in the development of hypothyroidism [6,9,12,13]. Similar results were seen in our study, where 8.3% (2/24) of individuals with neck radiation doses less than 45 Gy had hypothyroidism, while those receiving radiotherapy doses of more than or equal to 45 Gy showed an incidence of hypothyroidism of 18.48% (14/76), which was statistically not significant.

In our study, 74 patients received chemotherapy along with radiation as part of their treatment. Among patients who received chemotherapy, 18.91% (14/74) developed hypothyroidism, while 7.6% (2/26) of those who did not receive chemotherapy developed it. This difference was not statistically significant (p>0.05). Similarly, in the study by Posner et al. on head and neck cancer patients, they saw the effect of concurrent chemoradiotherapy on hypothyroidism and found that there was no association [9]. Similarly, Sinard et al. and Mercado et al. observed no increase in the incidence of hypothyroidism in patients with head and neck carcinoma who received chemotherapy as part of the treatment [10,14].

Time duration to develop hypothyroidism following radiotherapy varied widely. Fujiwara et al. reported 21 months (5-57 months) after radiotherapy as the median time to the onset of hypothyroidism [15]. Rønjom demonstrated that hypothyroidism is most likely to develop within the first two to three years after radiotherapy [16]. Koc and Capoglu observed a median time of 15 months for the development of clinical hypothyroidism and three months for subclinical hypothyroidism [17]. However, we found the median time duration to become hypothyroid after EBRT was 12 months, which was similar to the findings of Glatstein et al. [18]. The fact that conventional radiotherapy with cobalt 60 was utilized in our study could explain the variation in time to develop hypothyroidism.

There are a few possible limitations associated with our study, including non-randomized study design and convenience sampling might affect the generalizability. Also, the duration of follow-up was not long enough to thoroughly evaluate late toxicities. In spite of this, our study provides useful information regarding hypothyroidism after radiotherapy for head and neck cancers, which can be helpful in both treatment planning and follow-up. Considering the high incidence of hypothyroidism associated with neck radiation, we recommend that thyroid function be monitored as part of routine follow-up procedures among all cancer patients receiving neck radiation.

## Conclusions

Subclinical hypothyroidism and clinical hypothyroidism occur with considerable and poorly acknowledged frequency in cancer patients treated with radiotherapy to the neck region. The monitoring of thyroid function should become a part of routine follow-up procedures in all cancer patients who receive neck radiation as part of their treatment.
